# Impact Analysis of COVID-19 Pandemic on Hospital Reviews on Dianping Website in Shanghai, China: Empirical Study

**DOI:** 10.2196/52992

**Published:** 2024-07-02

**Authors:** Weixue Huo, Mengwei He, Zhaoxiang Zeng, Xianhao Bao, Ye Lu, Wen Tian, Jiaxuan Feng, Rui Feng

**Affiliations:** 1 Department of Vascular Surgery Shanghai General Hospital Shanghai Jiaotong University Shanghai China; 2 Department of Vascular Surgery Changhai Hospital Navy Medical University Shanghai China; 3 Vascular Surgery Department Ruijin Hospital Shanghai Jiaotong University School of Medicine Shanghai China

**Keywords:** patient satisfaction, physician-patient relationship, ChatGPT, patient concern, COVID-19

## Abstract

**Background:**

In the era of the internet, individuals have increasingly accustomed themselves to gathering necessary information and expressing their opinions on public web-based platforms. The health care sector is no exception, as these comments, to a certain extent, influence people’s health care decisions. During the onset of the COVID-19 pandemic, how the medical experience of Chinese patients and their evaluations of hospitals have changed remains to be studied. Therefore, we plan to collect patient medical visit data from the internet to reflect the current status of medical relationships under specific circumstances.

**Objective:**

This study aims to explore the differences in patient comments across various stages (during, before, and after) of the COVID-19 pandemic, as well as among different types of hospitals (children’s hospitals, maternity hospitals, and tumor hospitals). Additionally, by leveraging ChatGPT (OpenAI), the study categorizes the elements of negative hospital evaluations. An analysis is conducted on the acquired data, and potential solutions that could improve patient satisfaction are proposed. This study is intended to assist hospital managers in providing a better experience for patients who are seeking care amid an emergent public health crisis.

**Methods:**

Selecting the top 50 comprehensive hospitals nationwide and the top specialized hospitals (children’s hospitals, tumor hospitals, and maternity hospitals), we collected patient reviews from these hospitals on the Dianping website. Using ChatGPT, we classified the content of negative reviews. Additionally, we conducted statistical analysis using SPSS (IBM Corp) to examine the scoring and composition of negative evaluations.

**Results:**

A total of 30,317 pieces of effective comment information were collected from January 1, 2018, to August 15, 2023, including 7696 pieces of negative comment information. Manual inspection results indicated that ChatGPT had an accuracy rate of 92.05%. The F1-score was 0.914. The analysis of this data revealed a significant correlation between the comments and ratings received by hospitals during the pandemic. Overall, there was a significant increase in average comment scores during the outbreak (*P*<.001). Furthermore, there were notable differences in the composition of negative comments among different types of hospitals (*P*<.001). Children’s hospitals received sensitive feedback regarding waiting times and treatment effectiveness, while patients at maternity hospitals showed a greater concern for the attitude of health care providers. Patients at tumor hospitals expressed a desire for timely examinations and treatments, especially during the pandemic period.

**Conclusions:**

The COVID-19 pandemic had some association with patient comment scores. There were variations in the scores and content of comments among different types of specialized hospitals. Using ChatGPT to analyze patient comment content represents an innovative approach for statistically assessing factors contributing to patient dissatisfaction. The findings of this study could provide valuable insights for hospital administrators to foster more harmonious physician-patient relationships and enhance hospital performance during public health emergencies.

## Introduction

### The Tense Relationship Between Chinese Patients and Health Care Service Providers

In recent years, medical issues have been a highly focused and hot topic in China [1,2]. In 2016, 2017, and 2019, China experienced severe conflicts between patients and health care providers, including extreme cases of violence where doctors were harmed or even killed by patients or their family members [3,4]. The occurrence of this series of events highlights the challenging state of patient-health care service provider relationships in China. Moreover, the significance of these relationships cannot be understated. On the one hand, doctor-patient relationships have a profound impact. They influence patients’ satisfaction with health care services and their understanding of medical information. Additionally, these relationships affect patients’ behavior in response to illness and their adherence to prescribed medication, ultimately impacting their overall quality of life and health outcomes [5].

Therefore, it is important for us to improve the current state of patient-health care service provider relationships. Against this backdrop, the COVID-19 pandemic has further exacerbated the complexity of this issue [6]. The outbreak of SARS-CoV-2, causing COVID-19, has been an unprecedented event affecting all sectors of society, particularly the health care sector. The spread of the pandemic has resulted in a strained allocation of medical resources, potentially leading to shortages in certain health care services. Hospitals are required to prioritize patients based on varying levels of urgency and may need to implement isolation measures. These measures have the potential to impact the mental well-being of both health care workers and patients to varying degrees [7,8]. These factors could potentially lead to an even more strained relationship between Chinese patients and health care service providers.

### Web-Based Comments and ChatGPT

In the era of the internet, individuals are becoming increasingly accustomed to expressing their viewpoints on public web-based platforms and using public comments as a means to inform their own rational decision-making processes [9,10]. The field of health care is no exception, as previous research on quality management has indicated that, due to factors such as complex complaint processes and a lack of transparency in the resolution, only a third of patients opt to make a formal complaint when they encounter unsatisfactory service [11-13]. However, with the increasing prevalence of internet technology, public web-based platforms have provided patients with a more efficient platform to express their personal opinions. When faced with unsatisfactory service, the option of web-based reviews provides an alternative approach for patients to address their concerns. Through internet platforms, they can discuss their negative medical experiences and share them with others [14]. Research has demonstrated that the public dissemination of negative reviews has the potential to undermine or tarnish the image of physicians and amplify patient dissatisfaction with doctors or hospitals [15]. Undoubtedly, these comments also have the potential to exert an underlying influence on subsequent patients seeking medical care. Hence, the identification and documentation of negative patient comments, followed by targeted improvements, are of paramount importance in enhancing the quality of health care services and fostering positive physician-patient relationships [16]. Over the past year, considerable attention has been drawn to a novel AI model called ChatGPT due to its remarkable proficiency in accomplishing varied natural language tasks [17-21]. ChatGPT is a general large language model recently developed by OpenAI. Many studies have shown that ChatGPT has demonstrated impressive analytical and observational abilities, approaching or surpassing the threshold required to simulate the United States Medical Licensing Examination multiple-choice questions [17,22-25]. Leveraging the analytical capabilities of ChatGPT, this study aims to conduct content analysis and organize publicly available negative reviews to explore the specific reasons and composition of patients’ negative feedback.

Current research findings indicate that during the COVID-19 pandemic, the rating of doctor-patient relationships among patients (mean 4.18, SD 0.51) has increased compared to the prepandemic period (mean 3.86, SD 0.67), suggesting an improvement in the doctor-patient relationship. However, this study did not differentiate between specific types of hospitals. General hospitals and specialized hospitals may exhibit differences in doctor-patient relationships due to variances in the types of patients they treat. Additionally, when collecting rating data before the pandemic, this study relied on participants’ retrospective memory for scoring, which could introduce certain biases and deviate from the actual circumstances. Moreover, a patient’s experience during a hospital visit is composed of multiple factors; in addition to doctor-patient relationships, the quality of service provided by nurses and other hospital staff should also be taken into consideration. Even the cleanliness of the hospital environment and the quality of service offered by automated service equipment can affect the patient’s experience during a hospital visit [26,27].

This study goes beyond the conventional doctor-patient relationship. Instead, it starts by exploring and analyzing the patients’ public comments about the hospitals. By examining the assessments published on public platforms, we aim to investigate the impact of COVID-19 factors and hospital categorization differences on the health care experiences of patients in China, taking into account the perspectives of the patients. The research questions posed in this survey encompass three aspects. Research question (RQ) 1: During the pandemic, how have patient ratings for hospitals changed? RQ 2: Since the outbreak of the pandemic, what changes have occurred in the composition of negative reviews from patients about hospitals? RQ 3: What kind of negative feedback have different types of hospitals—such as children’s, maternity, and tumor hospitals—received, and what are the characteristics of the negative reviews for these various institutions?

## Methods

### Sample and Data Collection

**Figure 1:**
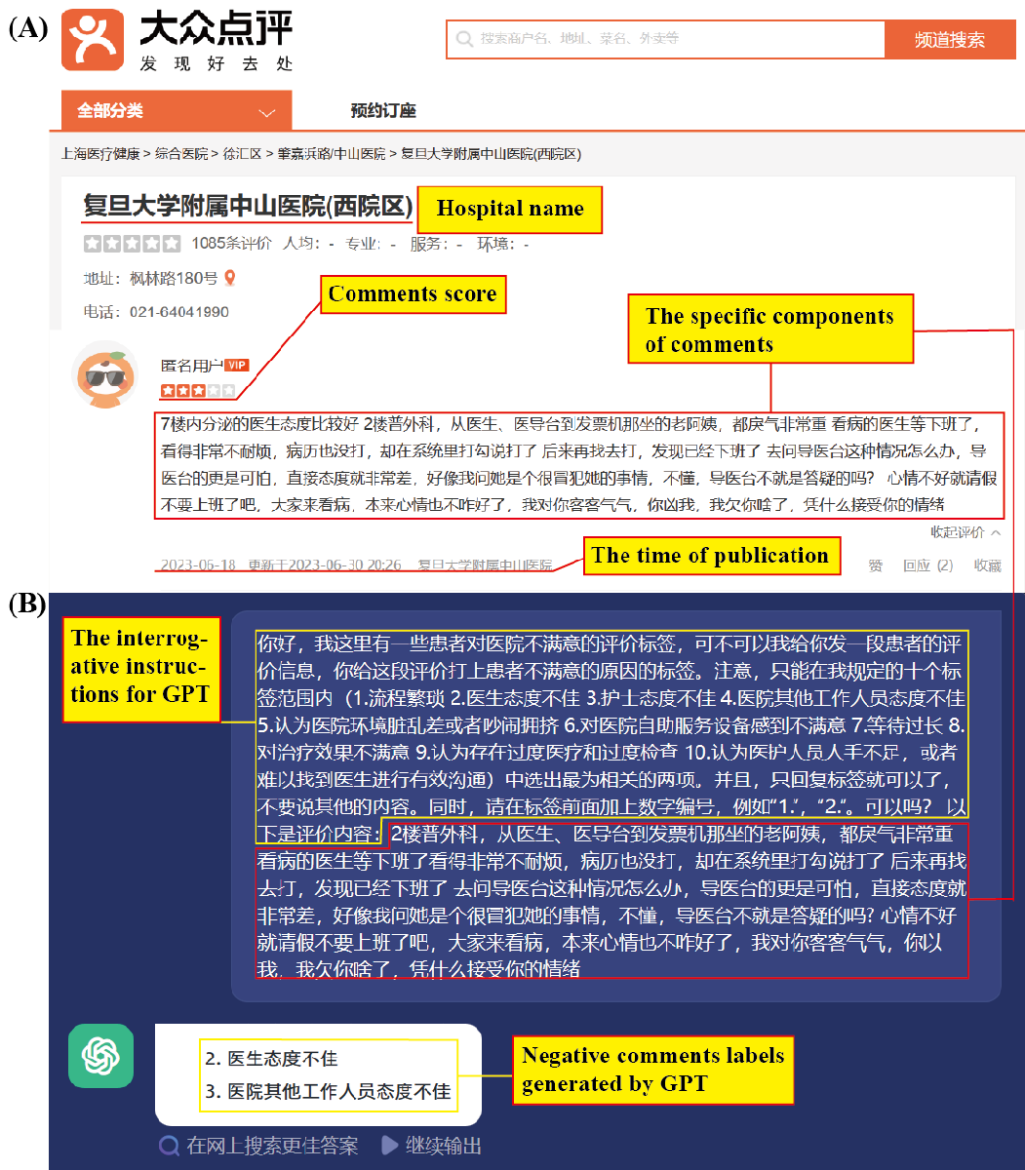
(A) Negative comment samples from the Dianping website. (B) ChatGPT handling demonstration.

This study relied on the open business review platform website Dianping [28]. Established in Shanghai in April 2003, this platform is the earliest independent third-party review website globally, with an active user base of 40.987 million people. The sample for this study consisted of a total of 88 hospitals, including those ranked in the top 50 nationally by Fudan University and those nominated in the Fudan University Specialist Hospital rankings for children’s hospitals, maternity hospitals, and tumor hospitals. The collection of comment information was limited to the period from January 1, 2018, to August 15, 2023. The collected content included the hospital’s region, type, name, date, score (ranging from 1 to 5), content, and user ID (Figure 1A). In order to ensure patient privacy, the user IDs were only used to judge whether the comment information met the requirements and do not appear in this paper.

### Comment Inclusion Criteria

It was necessary to conduct preliminary screening, labeling, and exclusion of meaningless comment content to ensure the quality of the included comment information. Comment information with a total character count less than 10 and comment information with fewer than 10 different Chinese characters was directly deleted. Comment information corresponding to user IDs that appeared more than 5 times underwent manual review to confirm its authenticity and validity before being included.

### Processing and Analysis of Comment Scores

Based on the comment dates, the comment information was categorized as follows: (1) before the pandemic (January 1, 2018, to April 30, 2018), (2) during the pandemic (January 1, 2020, to April 30, 2020), and (3) after the pandemic (January 1, 2023, to April 30, 2023). The Kruskal-Wallis test was used to compare the comment score data among the three categories: (1) before the pandemic, (2) during the pandemic, and (3) after the pandemic. Subsequently, pairwise Mann-Whitney *U* tests were conducted to compare the 3 groups, and the statistical analysis results were recorded. The significance level was set at *P*<.05 (2-tailed).

The comment information was categorized based on hospital location: (1) Shanghai region and (2) other regions. A pairwise Mann-Whitney *U* test was used to compare the comment score data between hospitals in the Shanghai region and hospitals in other regions, and the statistical analysis results were recorded. The significance level was set at *P*<.05 (2-tailed).

The comment information was also categorized based on hospital type: (1) general hospitals, (2) children’s hospitals, (3) maternity hospitals, and (4) tumor hospitals. A Kruskal-Wallis test was used to compare the comment score data among children’s hospitals, maternity hospitals, and tumor hospitals. Subsequently, pairwise Mann-Whitney *U* tests were conducted to compare the 3 groups, and the statistical analysis results were recorded. The significance level was set at *P*<.05 (2-tailed).

### Processing and Analysis of Comment Content

Three clinical experts (RF, JF, and ZZ) were initially assigned to randomly review 500 comments with scores less than or equal to 3. After a thorough discussion among the experts, a compilation of 10 reasons was derived. This compilation was named the hospital-patient factor (HPF).

Using ChatGPT, a comprehensive analysis and extraction of all comments with scores less than or equal to 3 was conducted. For each negative comment, up to 2 reasons behind the low scores were extracted from the patient’s feedback. The instructional prompts provided to ChatGPT were as follows (Figure 1B):

Hello, I have a collection of patient dissatisfaction comment tags regarding the hospital. I will provide you with a patient’s comment and you will assign appropriate tags indicating the reasons for their dissatisfaction. Please note that you can only select from a predefined set of ten tags. (HPF-A: Long waiting times ,HPF-B: Dissatisfaction with the hospital’s self-service equipment ,HPF-C: Dissatisfaction with the treatment effect ,HPF-D: Poor attitude of nurses ,HPF-E: Cumbersome medical treatment process ,HPF-F: Concerns about overtreatment ,HPF-G: Perception of understaffed medical personnel ,HPF-H: Poor hospital environment ,HPF-I: Poor attitude of doctors ,HPF-J: Poor attitude of other hospital staff) Please select the two most relevant tags. And please respond with only the tags, without any additional content. Here is the comment: “+ [comment content].”

After processing all the data, comments that could not be tagged with specific HPF labels by ChatGPT were deleted. A systematic sampling survey of the ChatGPT output was conducted. Out of every 100 comments, 10 were extracted and given to 3 clinical experts for manual review in order to assess the authenticity and validity of the ChatGPT results.

### Ethical Considerations

The data collected for this study consisted of deidentified information that has been publicly released on the web, adhering to ethical standards while fully respecting and safeguarding the rights of all participants. Furthermore, this study underwent a rigorous review and received approval from the Ethics Committee of Shanghai General Hospital (20240417094101248).

## Results

### Overview

**Figure 2:**
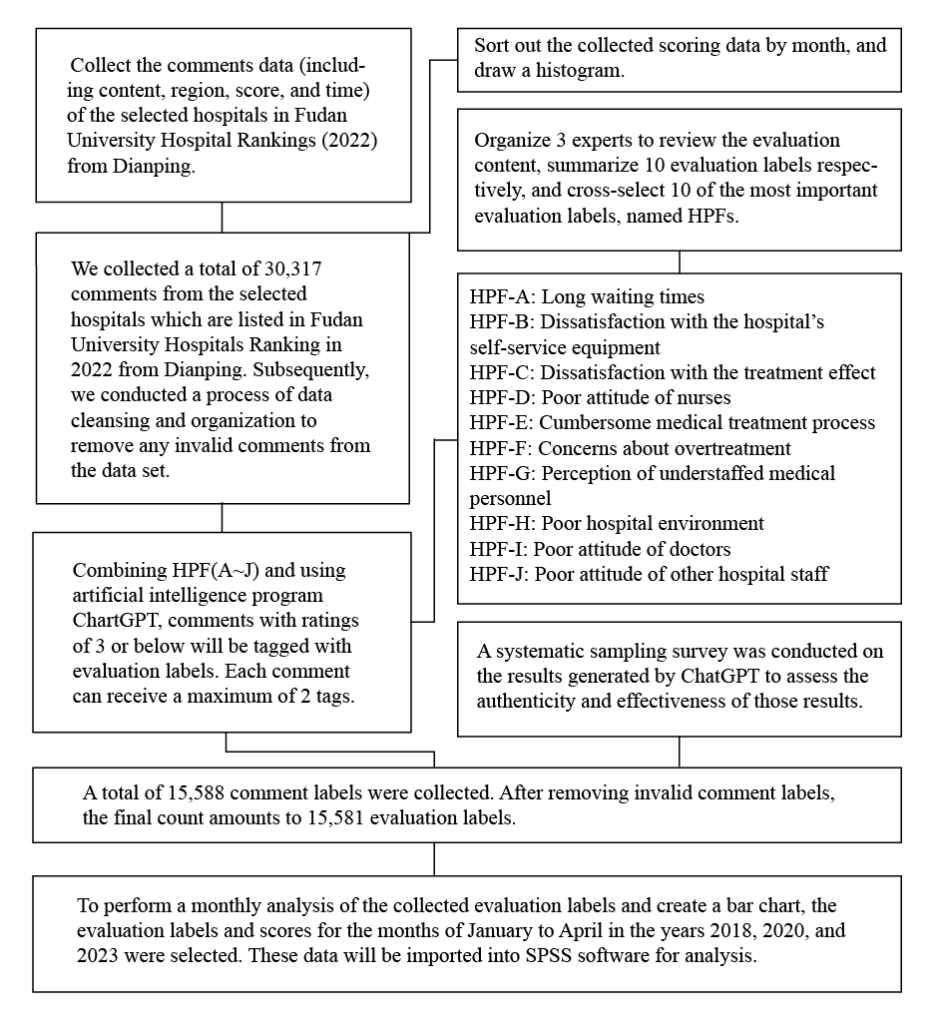
Flowchart depicting the process of data collection and organization in this study. HPF: hospital-patient factor.

The general flow of comment information from collection to processing is shown in Figure 2. In this study, a total of 32,085 pieces of comment data were gathered, and after careful screening, 1768 comments that did not meet the inclusion criteria were excluded. This resulted in a final collection of 30,317 valid comment records. (Multimedia Appendix 1). Among them, 7696 were categorized as negative comments (with scores less than or equal to 3). After undergoing processing through ChatGPT, the negative comments yielded a total of 15,588 HPF tags, and after further scrutiny, 7 invalid tags were removed.

**Figure 3:**
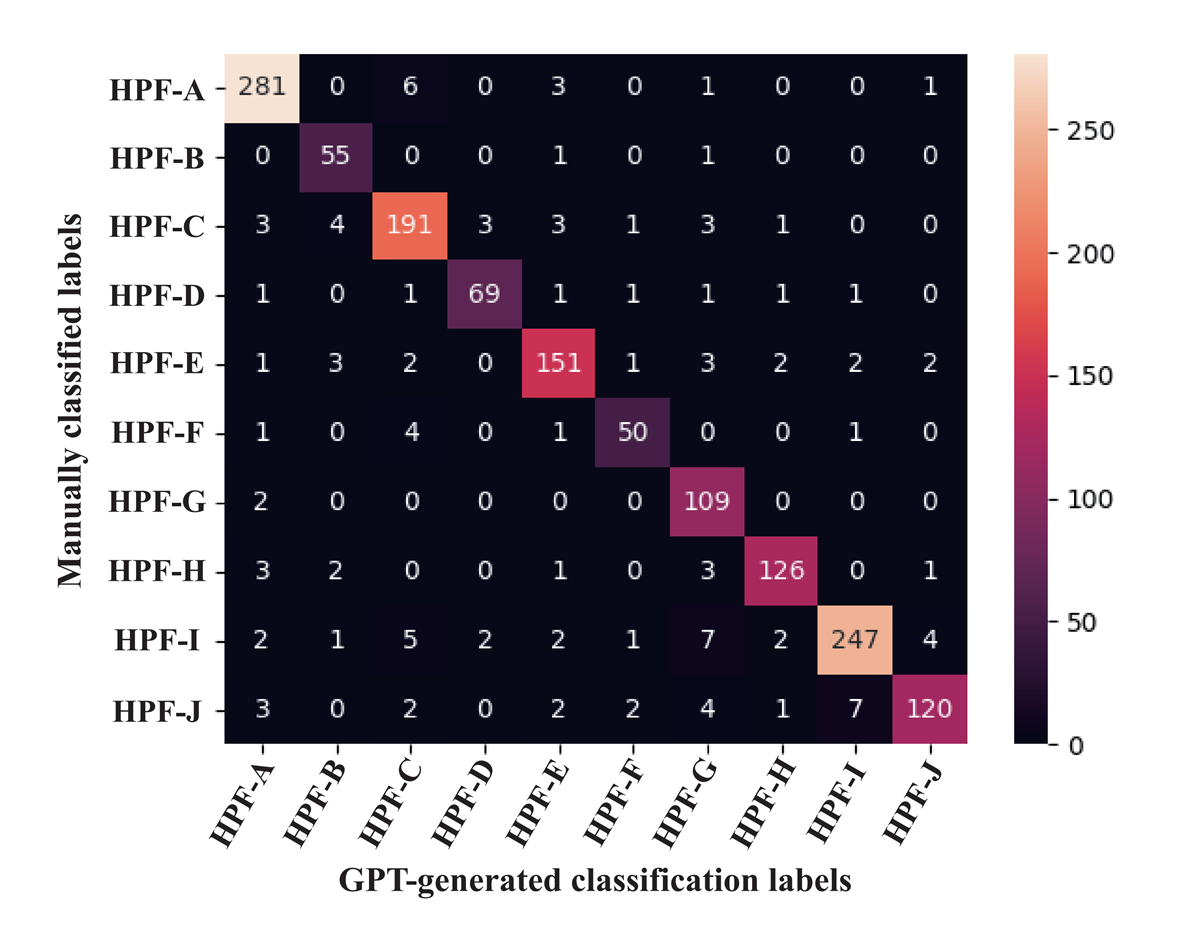
Confusion matrix: manual inspection of ChatGPT’s automatic classification results. HPF: hospital-patient factor.

A systematic random sampling survey was conducted on the remaining output results to confirm their authenticity and effectiveness. A total of 1519 labels were manually reviewed, and the results show that the accuracy of ChatGPT’s identifications reached 92.05%, and a confusion matrix for ChatGPT’s automatic classification assessment was plotted (Figure 3). Based on this, the performance of GPT in text classification was evaluated using manually encoded data. Among them, the macro *F*_1_-score was 0.914 (Table 1) [29,30]. Ultimately, 15,581 valid HPF tags were obtained. An overview of the collected data is presented in Table 2.

**Table 1:** Results of the GPT text classification performance evaluation.

Categorization of HPF^a^		Precision	Recall	*F*_1_-score
**Single HPF**				
	HPF-A	0.946	0.962	0.954
	HPF-B	0.846	0.965	0.902
	HPF-C	0.905	0.914	0.909
	HPF-D	0.932	0.907	0.921
	HPF-E	0.915	0.904	0.909
	HPF-F	0.893	0.877	0.885
	HPF-G	0.826	0.982	0.897
	HPF-H	0.947	0.926	0.937
	HPF-I	0.957	0.905	0.931
	HPF-J	0.938	0.851	0.892
**Aggregate**				
	Macro	0.911	0.919	0.914
	Micro	0.921	0.921	0.921

^a^HPF: hospital-patient factor.

**Table 2:** Overview of hospital grouping, number of hospitals and comment count.

Categorization of hospitals		Number of hospitals (n=88), n (%)	Number of comments (n=30,317), n (%)
**Area**			
	All hospitals	88 (100)	30,317 (100)
	Hospitals in Shanghai	19 (22)	14,511 (47,9)
	Hospitals in other areas	69 (78)	15,806 (52.1)
**Period**			
	Before the pandemic (January 2018 to April 2018)	88 (100)	1904 (6.3)
	During the outbreak of COVID-19 (January 2020 to April 2020)	88 (100)	1949 (6.3)
	After the pandemic (January 2023 to April 2023)	88 (100)	1016 (3.4)
**Type of** **hospital**			
	Children’s hospital	17 (19)	4910 (16.2)
	Maternity hospital	13 (15)	6685 (22.1)
	Tumor hospital	13 (15)	978 (3.2)

### Trend of Comment Scores in all Hospitals

**Figure 4:**
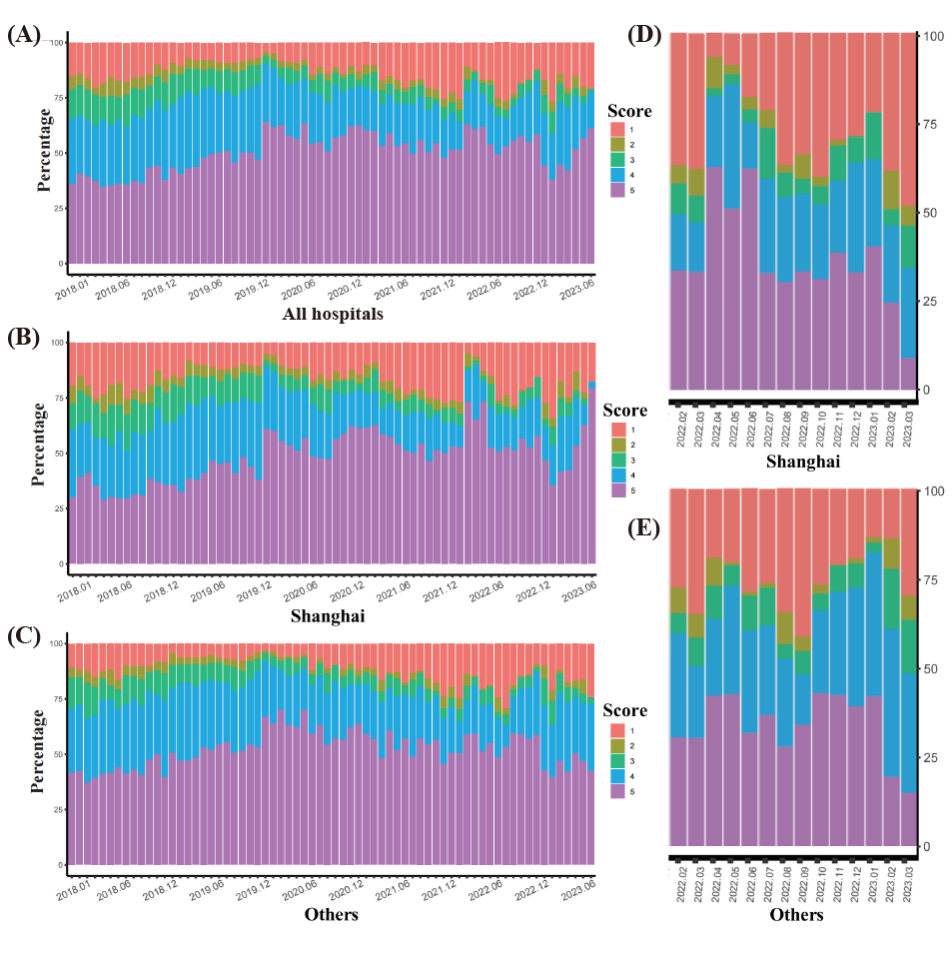
(A)-(C): Trend chart of score changes for all hospitals, hospitals in Shanghai, and hospitals in other areas from January 2018 to August 2023. (D) and (E): The M-shaped fluctuation graph of hospitals in Shanghai and hospitals in other areas.

The results indicate that the comment scores of all hospitals experienced a gradual increase from January 2018 to January 2019 and remained at a high level from February 2019 to November 2019. After the outbreak of the pandemic in December 2019, the comment scores of all hospitals rose again and reached a peak in February 2020. Subsequently, from March 2020 to March 2022, the comment scores of all hospitals gradually declined over a period of 2 years, reaching the lowest value since 2018 in March 2022. However, from April 2022 to January 2023, the comment scores experienced a sudden increase followed by a continuous decline. In October 2022, the comment scores sharply rose again, only to plummet after reaching their peak in January 2023. During the period from April 2022 to January 2023, the comment scores of all hospitals exhibited 2 drastic fluctuations, forming an M-shaped waveform. Subsequently, from January 2023 to August 2023, the comment scores remained at a lower level (Figure 4A).

### The Comment Scores Trend of Hospitals in Shanghai and Other Regions

The comment score trends of hospitals in Shanghai and other regions follow a similar pattern to the average score changes from all hospitals, encompassing a sequence of “gradual increase-maintenance-sudden increase-decline–M-shaped waveform-low score maintenance” (Figures 4B and 4C). During the M-shaped waveform period from April 2022 to January 2023, the M-shaped waveform in Shanghai was steeper and experienced more drastic fluctuations compared to other regions (Figures 4D and 4E). We conducted a separate analysis of the comment scores and content of comments for hospitals in Shanghai and other regions during the M-shaped waveform period (April 2022 to January 2023). We found that there were significant differences in the comment scores between hospitals in Shanghai and other regions during this period (*P*=.03). However, there was no significant difference in the composition of the comment content (*P=*.59; Table 3; Figures S1, S2, and S3 in Multimedia Appendix 2).

**Table 3:** Score forms for hospitals in the Shanghai region and other areas.

Characteristics		Values
Shanghai (score), mean (SD)		4.027 (1.49)
**Hospitals with each rank, n (%)**		
	1	74 (15)
	2	19 (4)
	3	24 (5)
	4	67 (14)
	5	297 (62)
	Total	481 (100)
Others (score), mean (SD)		3.874 (1.54)
**Hospitals with each rank, n (%)**		
	1	140 (19)
	2	21 (3)
	3	46 (6)
	4	132 (18)
	5	413 (54)
	Total	752 (100)
*P* value		.03

### The Comparison Between the Period Before and After the Outbreak of the Pandemic and During its Peak

**Figure 5:**
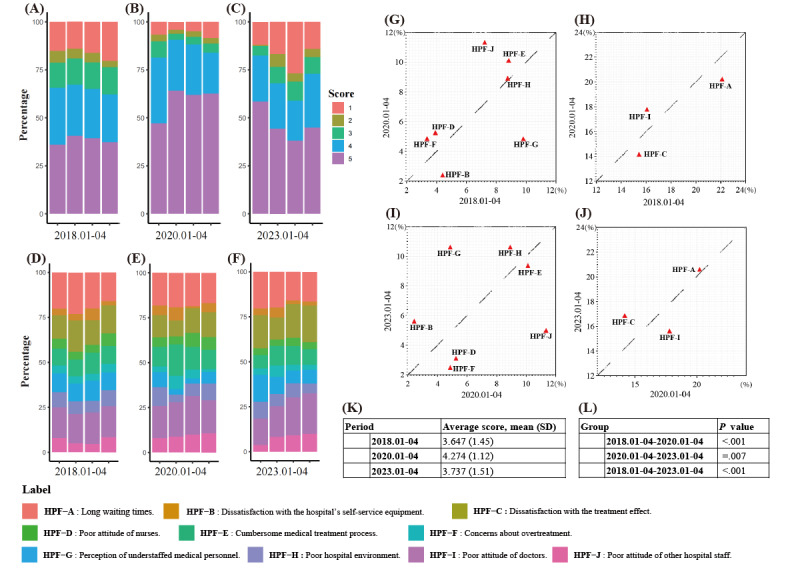
(A)-(C) Hospitals’ score diagrams for the months of January to April in 2018, 2020, and 2023. (D)-(F) Composition chart of hospital evaluation content for January-April in 2018, 2020, and 2023. (G) and (H) Comparison chart of hospital evaluation content composition for January to April in 2018 and 2020. (I) and (J) Comparison chart of hospital evaluation content composition for January to April in 2020 and 2023. (K) Average hospital scores for January to April in 2018, 2020, and 2023. (L) *P* value. HPF: hospital-patient factor.

Given the significant impact of the sudden outbreak of the pandemic on hospitals, we selected three periods for comparison. (1) Period A, before the outbreak (January 1, 2018, to April 30, 2018); (2) period B, during the outbreak (January 1, 2020, to April 30, 2020); and (3) period C, after the outbreak (January 1, 2023, to April 30, 2023). We compared the comment scores and analyzed the composition of negative feedback during these periods. There were clear significant differences in the scores between periods A, B, and C (*P*<.001). Pairwise Mann-Whitney *U* tests further confirmed significant differences among all groups (*P*=.007). The highest average score was observed in period B, at 4.274. Period A had an average score of 3.647, while period C had an average score of 3.737 (Figures 5A-5C, 5K, and 5L).

We conducted an analysis of the composition of negative feedback during periods A, B, and C, revealing significant differences among the 3 groups (*P*=.03). Pairwise comparisons using the chi-square test for 2 proportions showed no significant difference between periods A and C (*P*=.20). However, there was a relatively significant difference between periods A and B (*P*=.04), as well as between periods C and B (*P*=.07; Figures 5D-5F; Figure S4 in Multimedia Appendix 2). Period B, which corresponds to the outbreak period, exhibited a relatively higher proportion of negative comments categorized as HPF-D, E, F, I, and J (Figures 5G-5J). During periods A and C, which correspond to nonoutbreak periods, there was a relatively higher proportion of negative comments classified as HPF-B, G, and C.

### Comparison of Children’s Hospitals, Maternity Hospitals, and Tumor Hospitals

We compared the comment scores of these children’s hospitals, maternity hospitals, and tumor hospitals and found significant differences in the comment scores among these 3 distinct types of specialized medical institutions (*P*<.001). Furthermore, pairwise Wilcoxon rank-sum tests were conducted for each pair of data sets, demonstrating significant differences between any 2 groups (*P*<.001). Maternity hospitals had the highest average score, at 4.121. Children’s hospitals were next, with an average score of 3.812. Tumor hospitals had the lowest average score, at 3.572. Additionally, unlike hospitals of other types, tumor hospitals received lower comment scores during the outbreak compared to before the outbreak. On the other hand, children’s hospitals and maternity hospitals received the highest comment scores during the outbreak (Table 4; Figures S5, S6, and S7 in Multimedia Appendix 2).

**Table 4:** Score forms for children’s hospitals, maternity hospitals, and tumor hospitals.

Characteristics			Scores, mean (SD)
**Before the pandemic (January 2018 to April 2018)**			
	Children’s hospital	3.847 (1.48)	
	Maternity hospital	3.958 (1.31)	
	Tumor hospital	3.797 (1.42)	
**During the outbreak of COVID-19 (January 2020 to April 2020)**			
	Children’s hospital	4.003 (1.35)	
	Maternity hospital	4.461 (1.19)	
	Tumor hospital	3.404 (1.61)	
**After the pandemic (January 2023 to April 2023)**			
	Children’s hospital	3.757 (1.49)	
	Maternity hospital	4.241 (1.34)	
	Tumor hospital	3.394 (1.51)	

We conducted an analysis of the composition of negative comments for children’s hospitals maternity hospitals, and tumor hospitals, and found significant differences in the composition of comments among the 3 groups (*P*<.001). Further pairwise comparisons using chi-square tests revealed significant differences in the composition of negative comments between children’s hospitals and maternity hospitals, as well as between children’s hospitals and tumor hospitals (*P*<.001; Figures S8, S9, S10, and S11 in Multimedia Appendix 2).

**Figure 6:**
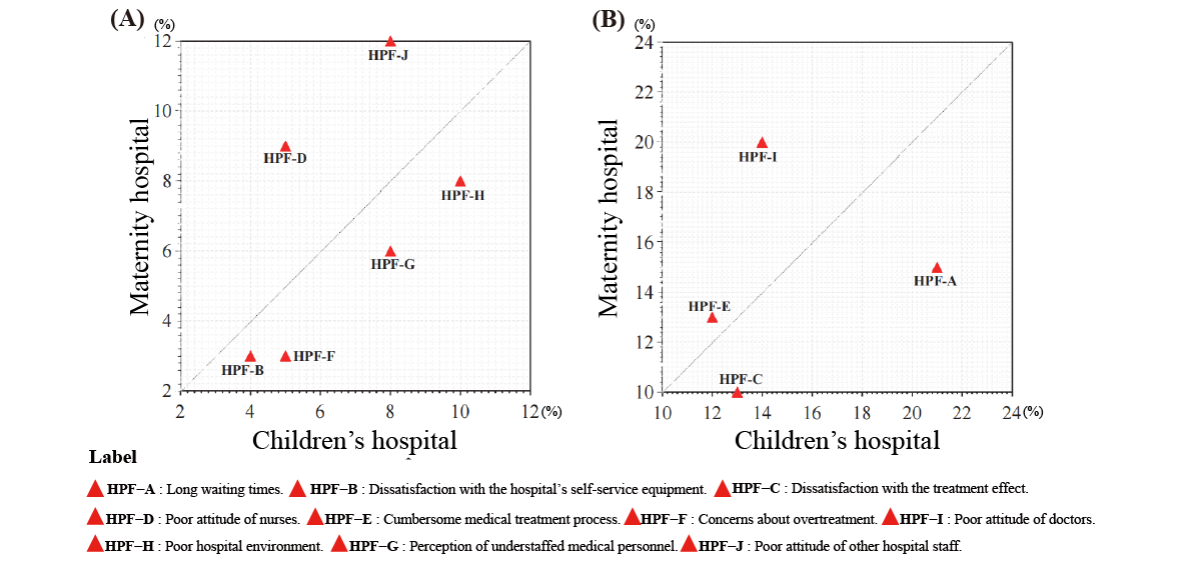
(A) and (B) Comparison chart of the composition of evaluation content between children’s hospitals and maternity hospitals. HPF: hospital-patient factor.

Compared to other hospitals, maternity hospitals tended to have a relatively higher proportion of negative comments related to HPF-D, J, and I, while children’s hospitals tended to have a relatively higher proportion of negative comments related to HPF-A, C, F, and G (Figure 6).

## Discussion

### Principal Findings

In this study, we compared the trends in comment scores and composition of comments exhibited by different hospitals in different regions and of different types during the periods before, during, and after the pandemic. We found that at the early stages of the COVID-19 outbreak, there was a certain increase in the patient satisfaction scores for hospitals (average score: 4.274; *P*<.001). However, as the pandemic continued, there was a gradual decline in the hospitals’ satisfaction scores from patients (average score: 3.737; *P*<.001). Moreover, during the pandemic, patients were more likely to express dissatisfaction with the attitudes of doctors, nurses, and other hospital staff.

In addition, in our survey of specialized hospitals, we discovered that, unlike other hospitals, patients at tumor hospitals tended to give lower scores during the pandemic (average score: 3.404; *P*<.001). This may be related to the anxiety that patients with cancer feel about their treatments. The negative feedback provided by patients at maternity hospitals and children’s hospitals also had their own distinctive characteristics.

### The Comment Score Trends for all Hospitals, Hospitals in Shanghai, and Hospitals in Other Regions

Our research demonstrates that during the COVID-19 pandemic in China, which spanned a 3-year period, patient comments on hospitals exhibited distinct characteristics across different stages. Existing findings indicate that during the peak of the outbreak (January to June 2020), the physician-patient relationship showed signs of improvement, reaching its optimal state in February 2020. Positive media coverage of health care workers, the adoption of telemedicine, and supportive health care policies had a significant and positive impact on the physician-patient relationship during the pandemic [6,31-33]. Physicians have perceived an increased level of trust from patients in their clinical practice compared to the past. For instance, patients voluntarily turn their heads away when sneezing and display greater respect toward doctors during conversations.

However, the general public does not share the belief that the physician-patient relationship has improved during the pandemic. According to a survey, 61% of the population expressed opposition to the idea of any improvement in the physician-patient relationship during this time [34]. The variations in attitudes and behaviors can be explained by several factors. First, the Chinese government has undertaken the entire cost of treating patients with COVID-19, relieving them of financial concerns and subsequently fostering improved attitudes toward seeking medical care and willingness to undergo treatment. This has contributed to a reduction in the sense of mistrust between doctors and patients [35,36]. Health care providers may perceive an improvement in the physician-patient relationship due to these factors. However, the general public, which is not affected by COVID-19, does not benefit from waived medical expenses. On the contrary, the scarcity of medical resources during the pandemic makes it even more challenging for the general population to seek medical care.

Positive media coverage and the positive impacts of health care policies during the pandemic may not necessarily be sustained in the long run [37,38]. Over a 2-year period from March 2020 to March 2022, patient ratings of hospitals gradually declined, reaching their lowest point in March 2022. Multiple factors contributed to this decline. According to Roubille et al [39], as the duration of the pandemic became prolonged, the confidence of the general public may have waned or suffered, ultimately leading to a deterioration in physician-patient relationships due to the adverse effects of COVID-19.

### M-Shaped Pattern

As shown in Figure 4A, during the period of April 2022 to January 2023, the comment scores of all hospitals experienced two significant fluctuations, forming a distinctive M-shaped pattern. Analyzing the timing, research indicates that the formation of the 2 peaks of the M-shaped pattern correlates with the outbreak in Shanghai in the first half of 2022 and the subsequent comprehensive relaxation of COVID-19 control measures in China toward the end of 2022. We have observed that these 2 incidents have caused varying degrees of strain on medical resources [40], while simultaneously leading to higher comment scores from patients toward hospitals. This finding aligns with previous research in this area [6,31-33].

As shown in Figures 4D and 4E, the M-shaped waveform formed during the outbreak in Shanghai from April to July 2022 was steeper, with the highest comment scores reached in April 2022. In contrast to the Shanghai region, hospitals in other areas exhibited a smoother M-shaped waveform, with scores lower than the peak scores in Shanghai. During this period, both Shanghai and other regions implemented the same health care policies, and the media coverage received by the public was consistent. In contrast, the situation in Shanghai is characterized by a more severe outbreak and a greater strain on medical resources.

Based on these observations, we speculate that, under similar circumstances, the scarcity of medical resources and the severity of the epidemic may lower patients’ expectations for medical outcomes. Consequently, they would value the precious medical resources even more, leading to an increase in comment scores and a decrease in negative reviews.

### Comparison Between the Outbreak and Before and After the COVID-19 Pandemic

We selected data from January to April 2020, as well as data from January to April 2018 and 2023, representing the periods of the pandemic outbreak, prepandemic, and postpandemic, respectively. The results reveal that hospitals received the highest comment scores during the outbreak, with an average score of 4.274. In comparison, the average scores for the prepandemic and postpandemic periods were 3.647 and 3.737, respectively. We have already discussed these findings in earlier sections (Figures 5A-5C, 5K, and 5L). Furthermore, through further analysis of the composition of negative comments during these 3 periods, we found certain differences between the prepandemic and pandemic outbreak periods, as well as the postpandemic and pandemic outbreak periods. During the pandemic outbreak, there was a relatively higher proportion of HPF-D, E, F, I, and J in negative comments, while HPF-B, G, and C showed a relatively lower proportion compared to the prepandemic and postpandemic periods (Figures 5D-5J; Figure S4 in Multimedia Appendix 2).

HPF-D, HPF-I, and HPF-J, respectively, represent patient dissatisfaction with nurses, physicians, and other hospital staff. The highest proportion of negative comments was “dissatisfaction with physician attitude.” For a long time, “dissatisfaction with physician attitude” has been a significant factor in the occurrence of doctor-patient disputes. These disputes not only directly lead to conflicts between doctors and patients but also erode the level of trust between them, resulting in a stalemate in the diagnosis and treatment process and affecting the quality and standard of health care [41,42]. The reasons for this situation are multifaceted, encompassing significant information asymmetry between doctors and patients, the authoritative status of doctors, poor doctor-patient communication, and a lack of empathy toward patients [43]. During the pandemic, the limited availability of medical resources has led to reduced communication time between doctors and each patient. Consequently, effective communication skills have become crucial in such circumstances. Numerous studies have demonstrated that fostering a positive doctor-patient communication environment can enhance patient trust, contribute to superior treatment experiences, and ultimately improve patient satisfaction and treatment outcomes [42-44]. Hospitals should also strengthen training in clinical and communication skills [44]. When dealing with critically ill patients, physicians must promptly communicate the prognosis to the patients. It is important to alleviate any unrealistic expectations the patients may have regarding the outcome and share the decision-making process with them in order to prevent medical disputes and the deterioration of the doctor-patient relationship [45,46].

Similarly, nurses and other hospital staff members are crucial components of the health care system. During the pandemic, there has been a notable increase in patients’ dissatisfaction with nurses and other staff members. Hospital administrators should prioritize their care and training to enhance service quality. Patient-centered nursing care can provide a better treatment experience for patients [47]. Moreover, there has been an increase in HPF-E during the pandemic, which emphasizes the need for hospitals to optimize complex health care processes to minimize negative patient experiences. This result reminds us that simplifying complicated medical procedures during a pandemic may help reduce the generation of negative emotions in patients.

### Differences Between Children’s Hospitals, Maternity Hospitals, and Tumor Hospitals

We selected 2 time points, December 2019, and November 2022, as reference periods to categorize the comment data into 3 stages: prepandemic, during the pandemic, and postpandemic. It is noteworthy that while most hospitals experienced an improvement in their comment scores during the pandemic, the scores for cancer hospitals instead showed a decline. From a rating of 3.797 before the pandemic, it decreased to 3.404 during the pandemic (Table 4; Figure S7 in Multimedia Appendix 2). This anomalous phenomenon may be attributed to the unique characteristics of patients with cancer. Compared to ordinary patients, patients with cancer often bear a heavier psychological burden, manifested through severe depressive and anxiety symptoms [48].

Furthermore, during the pandemic, the implementation of containment policies and the scarcity of health care resources may have significantly disrupted the diagnosis and treatment of patients with cancer. Research by Terashima et al [49] demonstrates that the COVID-19 pandemic, with its strain on health care resources, can result in delayed diagnoses, leaving patients with cancer already in advanced stages by the time they have the opportunity for diagnosis. The pandemic may also lead to treatment delays for patients with cancer, such as postponed surgeries due to circumstances beyond their control [49-51]. Given the circumstances described above, the impact of encountering the COVID-19 pandemic can be disastrous for patients with cancer who are in urgent need of diagnosis and treatment. Research by Ye et al [52] has elucidated that during the COVID-19 outbreak, patients with cancer may experience heightened psychological distress due to treatment delays. This suggests that the consequences for patients with cancer, when faced with the dual challenge of seeking timely diagnosis and navigating the pandemic, can be profound and deeply distressing [52,53]. This could be a contributing factor to the lower comment scores of cancer hospitals during the pandemic.

Therefore, for cancer hospitals, ensuring timely diagnosis and treatment for patients with cancer in the face of potential future outbreaks is crucial for enhancing health care service quality.

When analyzing the composition of negative comments, children’s hospitals and maternity hospitals exhibit prominent characteristics. The categories of HPF-A, C, F, and G in negative comments are relatively high in children’s hospitals (Figures 6A and 6B). These categories respectively represent “excessive waiting time,” “dissatisfaction with treatment effectiveness,” “perceived excessive medical procedures and examinations,” and “insufficient health care staff.” On one hand, the negative comments of “excessive waiting time” and “insufficient health care staff” authentically reflect the current shortage of pediatric doctors in China [54,55]. Based on the basic data from the Chinese Pediatric Medical Resources White Paper, China has approximately 260 million children aged between 0 and 14 years, but currently, there are only around 100,000 pediatric doctors nationwide. On average, each pediatric doctor is responsible for the care of 2000 children. This indicates a shortage of at least 200,000 pediatric doctors in China [56]. The shortage of pediatric doctors is a significant factor contributing to negative comments about children’s hospitals. Furthermore, the negative comments of “dissatisfaction with treatment effectiveness” and “perceived excessive medical procedures and examinations” reflect the anxiety and lack of trust that parents of sick children commonly experience toward doctors [55].

Given the aforementioned context, addressing the shortage of pediatric doctors and alleviating the negative emotions of parents of sick children have emerged as 2 crucial focal points for enhancing the quality of health care services in children’s hospitals. Focusing on the aforementioned dimensions, hospital administrators should prioritize training programs for pediatric doctors. Additionally, it is essential to enhance training for pediatric doctors and improve their salary levels without compromising their passion and dedication to caring for children [56,57].

When examining the negative comments of obstetric and gynecological hospitals, it becomes apparent that the proportions of HPF-D, J, and I are relatively high (Figures 6A and 6B). These refer respectively to “poor nurse attitude,” “poor doctor attitude,” and “poor attitude of other hospital staff.” It is intriguing to observe that negative assessments in the field of obstetrics and gynecology mainly revolve around “poor attitude.” This phenomenon can perhaps be explained from 2 perspectives. On one hand, during the outbreak of a pandemic, the number of patients has increased significantly, while the number of doctors has not kept pace. As a result, the amount of time and energy they can allocate to each patient is either reduced or can only be sustained by extending their working hours. A study revealed that 73% of obstetrics and gynecology practitioners work over 50 hours per week. In such long and intense work environments, the occupational burnout rate in the field of obstetrics can even reach 56.6% [58]. This will make obstetricians feel irritable and reduce their patience with the objects they serve or contact [59]. On the other hand, due to the abnormal hormones in pregnant women, fear of childbirth, and serious anxiety, these will make pregnant women more sensitive and uneasy [60-62].

In conclusion, in order to provide a positive medical experience for pregnant women and reduce the likelihood of negative comments, hospital administrators can address the following 2 issues: first, they can decrease the working hours of each health care professional, increase the number of specialized health care personnel, and improve the salary and benefits for health care workers. This will help alleviate the occupational burnout among obstetricians and gynecologists. Second, timely psychological interventions should be provided for pregnant women with a fear of childbirth, including evidence-based treatments such as psychoeducation, cognitive restructuring, and relaxation exercises. These interventions are believed to contribute to reducing the rate of cesarean section deliveries and enhancing overall satisfaction with the childbirth experience [63,64].

### The Limitations of This Study

This study has certain limitations. First, the selection of hospitals primarily focused on those with high national rankings, which are mainly located in economically developed cities. Therefore, the conclusions may not be applicable to hospitals in smaller cities. Second, unlike conventional surveys, this study only captured publicly available patient comment information, thus lacking demographic data on the individuals who provided these comments. Third, it was not possible to completely eliminate the possibility of some negative comments being maliciously posted by competitors as false information.

### Conclusion

The COVID-19 pandemic has some association with patient comment scores. In this study, comment scores of general hospitals, children’s hospitals, and maternity hospitals increased during the pandemic, excluding tumor hospitals. The scores and content of patient comments varied among different specialized hospitals. Analyzing patient comment content using ChatGPT is an innovative method for identifying factors contributing to patient dissatisfaction and provides a user-friendly approach. This study has certain reference value for hospital administrators in establishing harmonious relationships between health care providers and patients and in improving hospital performance during public health emergencies.

## Appendix

Multimedia Appendix 1 Original data: 30,317 pieces of hospital review information collected from the Dianping website.

Multimedia Appendix 2 Supplementary figures.

## Abbreviations

HPF: hospital-patient factor
RQ: research question

## Data Availability

All data generated or analyzed during this study are included in this published article and its supplementary information files.
