# Supplementary material for: Impact Analysis of COVID-19 Pandemic on Hospital Reviews on Dianping Website in Shanghai, China: Empirical Study
Source: J Med Internet Res. 2024 Jul 2;26:e52992. doi: 10.2196/52992 (PMC11252617; doi:10.2196/52992)
Supplement: Multimedia Appendix 2 [file jmir_v26i1e52992_app2.pdf]

S1

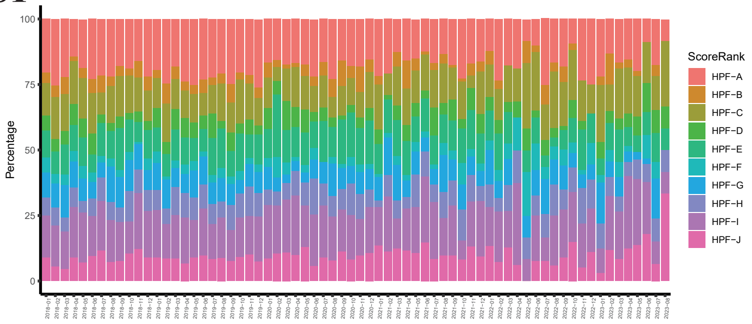

Shanghai

S2

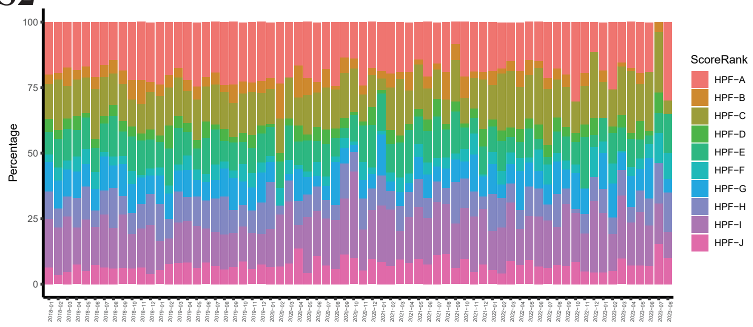

Others

S3

City/Label	HPF-A	HPF-B	HPF-C	HPF-D	HPF-E	HPF-F	HPF-G	HPF-H	HPF-I	HPF-J	Total	P-Value
Shanghai	*43*	*7*	*37*	*11*	*20*	*17*	*24*	*22*	*33*	*22*	236	*P=.596*
Others	*68*	*18*	*81*	*18*	*24*	*22*	*37*	*34*	*76*	*32*	410	
Total	111	25	118	29	44	39	61	56	109	54	646	

S4

Stage/Label	HPF-A	HPF-B	HPF-C	HPF-D	HPF-E	HPF-F	HPF-G	HPF-H	HPF-I	HPF-J	Total	Group	P-Value
2018.01-04	*257*	*55*	*195*	*73*	*136*	*57*	*139*	*104*	*228*	*88*	1332	2018-2020	*P=.038*
2020.01-04	*107*	*28*	*75*	*37*	*71*	*20*	*37*	*52*	*112*	*56*	595	2018-2023	*P=.188*
2023.01-04	*109*	*19*	*111*	*25*	*62*	*24*	*53*	*44*	*126*	*51*	624	2020-2023	*P=.069*
Total	473	102	381	135	269	101	229	200	466	195	2551	2018-2020-2023	*P=.033*

S5

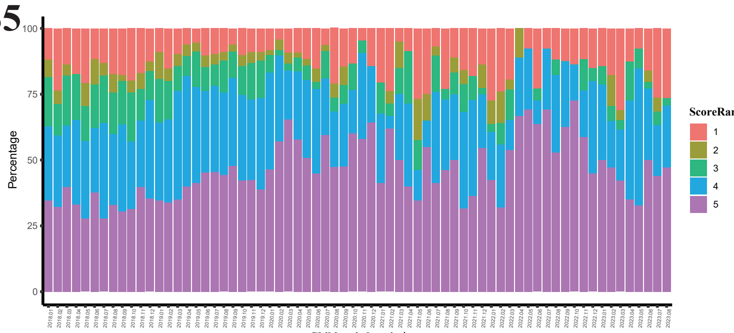

Children's hospital

S6

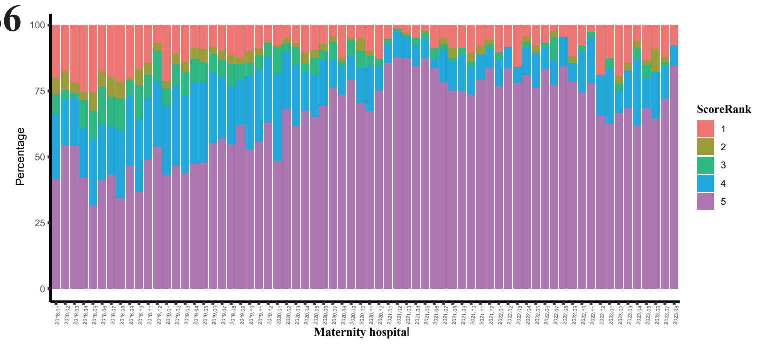

Maternity hospital

S7

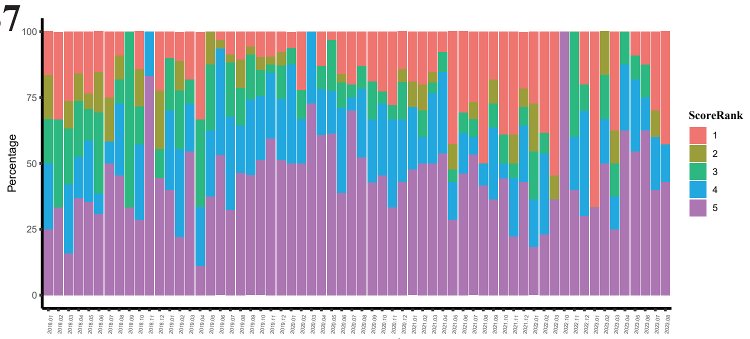

Tumor hospital

S8

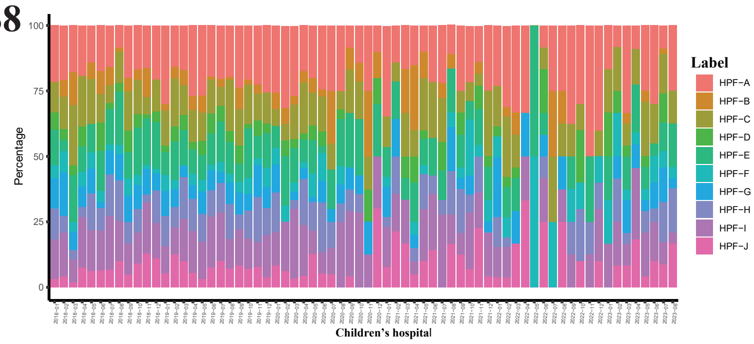

Children's hospital

S9

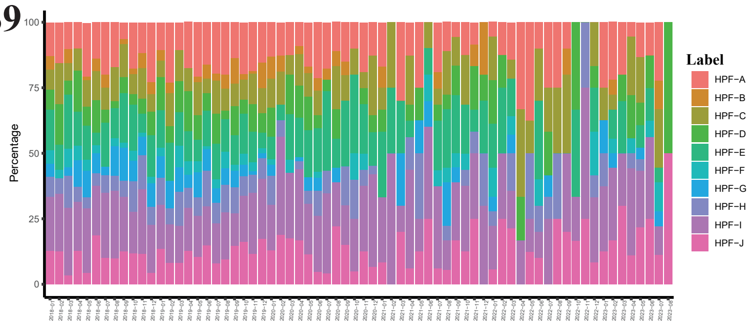

Maternity hospital

S10

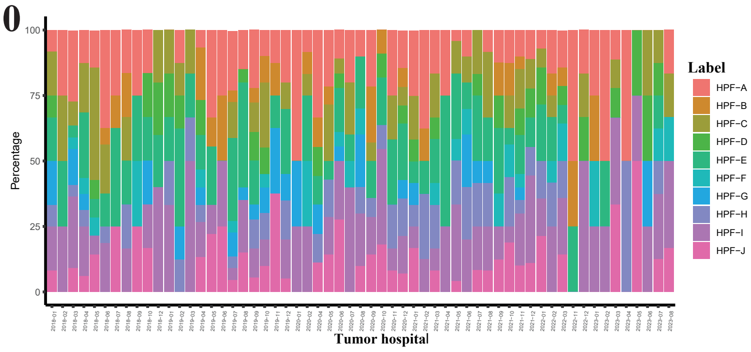

Tumor hospital

S11

Hospital/Label	HPF-A	HPF-B	HPF-C	HPF-D	HPF-E	HPF-F	HPF-G	HPF-H	HPF-I	HPF-J	Total	Group	P-Value
Children's Hospital	*582*	*116*	*362*	*127*	*345*	*128*	*213*	*286*	*398*	*217*	2774	CH-MH	*P<.001*
Maternity Hospital	*380*	*79*	*254*	*213*	*331*	*86*	*141*	*190*	*502*	*295*	2471	CH-TH	*P<.001*
Tumour Hospital	*102*	*30*	*72*	*33*	*107*	*27*	*32*	*55*	*116*	*66*	640	MH-TH	*P=.021*
Total	1064	225	688	373	783	241	386	531	1016	578	5885	CH-MH-TH	*P<.001*

Multimedia Appendix 2

S1-2:From January 2018 to August 2023, the distribution diagram of Hospitals in Shanghai and Hospitals in other areas with different monthly scores.

S3:Categorization of hospital evaluations and validity verification forms in both the Shanghai region and other areas(2022.04-2023.01).

S4:Classification and validity assessment forms for hospital evaluations from January to April in 2018, 2020, and 2023.

S5-7:Illustrative graph depicting the rating situation of children's hospitals, maternity hospitals and tumor hospitals.

S8-10:Illustrative diagram depicting the composition of evaluations for children's hospitals, maternity hospitals and tumor hospitals.

S11:Classification and validity assessment forms ffor children's hospitals, maternity hospitals and tumor hospitals.

CH: Children's Hospital;**MH:**Maternity Hospital;**TH:**Tumour Hospital.
